# Smoking as a Crucial Independent Determinant of Stroke

**DOI:** 10.1186/1617-9625-2-2-67

**Published:** 2004-06-15

**Authors:** Seana L Paul, Amanda G Thrift, Geoffrey A Donnan

**Affiliations:** 1 National Stroke Research Institute, Austin Health, Heidelberg West, Victoria 3081, Australia

## Abstract

**Background:**

Although smoking is known to be powerful risk factor for other vascular diseases, such as cardiac and peripheral vascular disease, only relatively recently has evidence for the role of smoking in the development of stroke been established. The reasons for this advance lie in the acknowledgement that stroke is a heterogeneous disease, in which its subtypes are associated with different risk factors. Furthermore, improvements in the stringency of epidemiological studies and the greater use of CT scanning have enabled the role of smoking in the development of stroke to be elucidated.

**Summary of review:**

This is a qualitative examination of high quality epidemiological studies in which the role of smoking and passive smoking, as a risk factor for cerebral infarction, intracerebral haemorrhage and subarachnoid haemorrhage, is examined. In addition, the pathological mechanisms by which smoking or passive smoking may contribute to the development of stroke are reviewed.

**Conclusion:**

Smoking is a crucial independent determinant of cerebral infarction and subarachnoid haemorrhage, however its role in intracerebral haemorrhage is unclear. Although studies are limited, there is evidence that exposure to passive smoking may also increase the risk of stroke. Smoking appears to be involved in the pathogenesis of stroke via direct injury to the vasculature and also by altering haemodynamic factors within the circulation. Importantly, smoking is modifiable risk factor for stroke. Therefore, the encouragement of smoking cessation may result in a substantial reduction in the incidence of this devastating disease.

## Introduction

Although smoking is an established and potent risk factor for other vascular diseases, such as cardiac and peripheral vascular disease [1,2], the evidence for its status as a risk factor for stroke has been gained only in the last 20 years [3-33]. That it took so long to establish smoking as a risk factor for stroke might be largely attributed to three main factors. First, epidemiological studies have become more stringent over the past two decades, with methodology being refined to reduce bias associated with ascertainment of both cases and controls. Second, the purpose of many of the earlier studies was not to determine whether smoking was a risk factor for stroke, but was mainly a result of post-hoc analyses. Consequently the methodology was more likely to include means of inadvertently choosing inappropriate samples for comparison. Finally, stroke is a heterogeneous condition, and it is only since the introduction of CT scanning that an accurate diagnosis of stroke and its subtypes has been possible. Previously, it is possible the epidemiological studies included other non-stroke conditions that may have been of nonvascular origin. Moreover, it is also possible that the differing aetiologies of cerebral ischaemia and cerebral haemorrhage may have resulted in a diminution of risk when these stroke subtypes were combined into an overall "stroke" group. Because of this possibility, we will discuss the association between smoking and its subtypes (cerebral infarction, subarachnoid and cerebral haemorrhage) separately. In addition, the studies that will be discussed are those that have a high rate of CT for accurate classification of these subtypes. This review is intended as a qualitative overview of the key literature regarding the association between smoking and stroke, and is not a detailed meta-analysis.

## Cerebral Infarction

Cerebral infarction is the most common subtype of stroke, accounting for more than 70% of all strokes [34]. Over the past few decades cigarette smoking has been established as a major risk factor for the development of cerebral infarction [11-13,17-19,21,23,25,28,35]. In a meta-analysis of 22 studies of the association between cigarette smoking and stroke prior to 1988, Shinton and Beevers found that the relative risk for cerebral infarction associated with smoking was 1.92 (95% CI, 1.71–2.16) [28].

The relationship between smoking and cerebral infarction has been confirmed in more recent case-control studies from the U.S., Australia, U.K., Scandinavia, and Russia (Table 1) [10-13,17,20,23,25,35]. In a study among more than 400 patients in Australia, Donnan and colleagues found that the risk of cerebral infarction due to smoking was substantially higher than in the previous meta-analysis [10]. In this study, which included adjustments for the potentially confounding effects of hypertension, high cholesterol, previous myocardial infarction, alcohol consumption and oral contraceptive use, the relative risk of cerebral ischaemia associated with current smoking was 3.6 (95% CI, 2.5–5.9). Similar results were found by Gorelick and colleagues in the United States and by Gill and colleagues in the United Kingdom [12,13]. In the latter case-control study, which included 368 cases of CI and 573 controls, the adjusted relative risk of CI with smoking was 3.19 (95% CI, 1.8–5.5) among men and 2.3 (95% CI, 1.2–4.2) among women after adjustment for confounding variables such as age, social class, hypertension and alcohol consumption.

**Table 1:** Smoking and the risk of cerebral infarction, summary of studies

	**No. CI cases (No. in cohort)**	**No. Controls**	**Smoking Status**	**RR/OR**	**95% CI**	**Adjustments†**	**Matching**
**Case-Control Studies**							
Donnan et al. [10] Melbourne, Australia, 1989	422	422	Never smoker	1.0		Hypertension, High cholesterol, MI, alcohol consumption, oral contraceptive use.	Age, sex, neighbourhood
			Ex smoker	2.0	1.3–3.2		
			Current smoker	3.6	2.2–5.9		
							
Gorelick et al [13] Illinois, U.S. 1989	205	410	0 (pack-years)	1.00		Hypertension, and alcohol consumption.	Age, race, sex and method of hospital payment
			1–32 (pack years)	2.48	1.43–4.29		
			≥ 33 (pack-years)	5.60	3.17–9.88		
							
Gill et al [12] British Midlands, U.K 1989	368	573	Nonsmokers	1.00		Age, race, social class, alcohol consumption and treatment of hypertension.	
			Current (Male)	3.19	1.8–5.5		
			Current (Female)	2.26	1.2–4.2		
							
Love et al. [23] Iowa, U.S. 1990	181 15–45 years	307	Current Smoker	1.43	0.98–2.11	No adjustments.	Age, gender, hospital admission date, and county of residence.
			Past Smoker	0.64	0.25–1.69		
			Per cigarette/day	1.01	1.00–1.03		
			Per pack-year	1.04	1.02–1.05		
							
Ellekjær et al [35] North Trøndelag County, Norway 1992.	163	567	Never smoker	1.00		Diabetes, previous myocardial infarction, previous stroke, and systolic blood pressure.	Sex, year of birth, and local government area.
			Current Smoking	1.72	0.85–3.51		
			Prior Smoking	2.54	1.22–5.29		
							
Lidegaard et al. [20] Denmark, 1993	320 females 15–44 years	1198	Never smoker	1.0		Age, use of oral contraceptives, years of schooling	
			Former smoker	0.6	0.4–0.9		
			Smoking ≤ 10/day	1.6	1.1–2.4		
			Smoking >10/day	1.5	1.1–2.0		
							
Jamrozik et al. [17] Perth, Australia, 1994	360	518	Never Smoker	1.00		Alcohol, hypertension, diabetes, previous stroke or TIA, previous MI, adding salt to food, fish consumption > 2 per month.	Sex and age.
			Ex-smoker	0.49	0.31–0.81		
			Current (1–20/day)	1.45	0.81–2.68		
			Current (≥ 21/day)	4.92	1.90–12.7		
							
Petitti et al [25] California, US 1996	144 females 15–44 years	774	Never	1.00		No adjustments.	Year of birth, location of facility.
			Past	0.94	0.55–1.59		
			Occasional	1.38	0.42–4.56		
			Current	2.66	1.65–4.30		
							
Feigin et al [11] Novosibirsk, Russia 1998	237	237	Non smoker	1.00		Hypertension, LVH, IHD, mitral valve disease, and BMI.	Age and sex.
			Current	2.20	0.99–4.78		

**Cohort Studies**							

Kawachi et al [18] Nurses' Health Study, U.S., 1993.	275 (117006 females)	Rest of stroke free cohort	Never Smokers	1.00		Age, hypertension, diabetes, BMI, high cholesterol, oral contraceptive use, HRT, age of smoking commencement, and follow-up period.	
			Ex-smokers	1.27	0.85–1.89		
			Current Smokers	2.53	1.91–3.35		
			1–14/day	1.83	1.04–3.23		
			15–24/day	3.57	2.36–5.42		
			25–34/day	2.73	1.49–5.03		
			≥ 35/day	3.97	2.09–7.53		
							
Benfante et al. [36] Hawaii, 1994	226	5567 (rest of cohort)	Non smokers	1.0			
			Current Smokers	2.30	1.8–3.0		
							
Lee et al. [21] Taiwan, 1995	115 (2600, > 65 years)	Rest of Cohort	Never smokers	1.00		Age, sex, hypertension, diabetes, and alcohol.	Prevalence Study
			Current Heavy (>20 per day)	1.72	1.00–2.96		
							
Kurth et al. [19] Physicians' Health Study, US, 2003	913 (22022 Males)	Rest of cohort	Never Smokers	1.00		Age, alcohol, exercise, parental history of MI <60 yr, and randomized treatment group.	
			Ex-Smokers	0.99	0.86–1.14		
			Current Smokers				
			<20/day	1.56	1.03–2.37		
			≥ 20/day	2.25	1.80–2.81		

CI, cerebral infarction; RR, relative risk; OR, odds ratio; 95% CI, Confidence Interval; †adjustments refer to adjustments made in multivariate analyses. MI, myocardial infarction; TIA, transient ischaemic attack; LVH, left ventricular hypertrophy; IHD, ischaemic heart disease; BMI, body mass index; HRT, hormone replacement therapy.

Similar results have been obtained in cohort studies in the United States and Taiwan [18,19,21,36]. In the U.S. Physicians' Health Study, Kurth and colleagues found that current smoking was associated with a twofold increase in the risk of ischaemic stroke (RR 2.11, 95% CI, 1.72–2.60) [19]. Those smoking <20 cigarettes/day were found to have a 1.6–fold increase in the risk of ischaemic stroke (RR, 1.56, 95% CI, 1.03–2.37) and this increased to 2.25 (95% CI, 1.80–2.81) among those smoking ≥ 20 cigarettes/day when compared to never smokers. These relative risks were adjusted for the confounding effects of age, alcohol consumption, exercise and parental history of myocardial infarction less than 60 years of age. In a similar study conducted in a large cohort of female nurses, Kawachi and colleagues found that current smokers were at a significantly greater risk of CI (RR 2.53, 95% CI, 1.91–3.35) [18]. Importantly, these investigators found a dose-response relationship between the level of smoking and cerebral infarction (p = 0.03). Those smoking 1–14 cigarettes/day had an adjusted RR of 1.8 (95% CI, 1.04–3.23), with the risk increasing to 3.97 (95% CI, 2.09–7.53) for women smoking ≥ 35 cigarettes/day., Together, these studies provide evidence that smoking increases the risk of cerebral infarction in both men and women, with the risk being greater in those who smoke more.

The use of pack-years by investigators to determine the dose of lifetime exposure to cigarettes was used in some studies [13,23]. This is a combination of both the number of cigarettes smoked per day and the duration of smoking (where a pack is defined as 20 cigarettes per day and this is multiplied by the number of years to calculate pack-years). Love and colleagues reported that the risk of cerebral infarction increased by 4% per pack-year smoked. In support of these findings, Gorelick and colleagues found that individuals who had been smoking for 1–33 pack-years had an odds ratio (OR) for cerebral infarction of 2.48 (95% CI, 1.43–4.29). This increased to an OR of 5.60 (95% CI, 3.17–9.88) in those smoking more than 33 pack-years. This provides evidence that there is an increased stroke risk with increased pack-years of smoking.

The difference in the definition used for exposure is an important issue. Measuring pack-years provides information on both the quantity of cigarettes smoked and the duration of this level of smoking. Thus it could be that either or both of these factors contribute to the increased stroke risk. It would be useful to determine whether one pack per day for 20 years produced a stroke risk similar to that of two packs per day for 10 years (with both equating to 20 pack-years). However, this type of information relies heavily on participant recall. Such accurate recall may be challenging, especially for individuals with cognitive deficits following stroke. Partly because of the difficulties outlined, it remains to be established whether it is the quantity or the duration of smoking that results in the increased risk of cerebral infarction.

The effect of cessation of smoking on the subsequent development of cerebral infarction is important in determining a causal relationship between this disease and smoking. In men, Kurth and colleages found that past smokers had a risk of cerebral infarction comparable to that of never smokers (RR 0.99, 95% CI, 0.86–1.14) [19]. Kawachi and colleagues provided further evidence for a return to never smoking risk levels among those who cease smoking [18]. They examined the number of years since quitting smoking (<2 years, 2–4 years, 5–9 years, 10–14 year and >15 years) on the risk of cerebral infarction in women. Current smokers were used as reference for the reduction in risk. At two years after quitting, the risk of stroke was reduced by 46% (RR 0.54, 95% CI, 0.26–1.55). This equated to 80% of the benefit of smoking cessation and demonstrating the immediate benefits of smoking cessation. Furthermore, the relative risk of ischaemic stroke among former smokers returned to the level of never smokers during the interval between 2 and 4 years following smoking cessation.

Similar results have been reported from case-control studies. In studies conducted in the U.S. [23,25], ex-smokers were found to have a risk of cerebral infarction similar to that of never smokers (RR 0.94, 95% CI, 0.55–1.59) and (0.64 95% CI, 0.25–1.69) for each of these studies respectively. Although it is possible that ex-smokers may reduce their risk of cerebral infarction simply by ceasing smoking, other lifestyle changes made at the time of smoking cessation may contribute to a reduction in cerebral infarction risk.

## Subarachnoid Haemorrhage

Subarachnoid haemorrhage (SAH) accounts for only about 2–9% of strokes [37], but is associated with mortality of between 39% and 58% at one year and significant morbidity [34,38]. This form of stroke tends to occur at younger ages than other forms of stroke [34]. The contribution of cigarette smoking to this devastating disease has been reported by numerous investigators [6,12,16,18,19,26,28]. In a meta-analysis of 10 studies of the association between smoking and SAH, the relative risk of SAH among current smokers was 2.93 (95% CI, 2.48–3.46) [28]. More recently, Kurth and colleagues found that among current smokers the age-adjusted RR for SAH was 3.61 (95% CI 1.54–8.50; Table 2) [19]. This study involved more than 22,000 male physicians and included 31 cases of SAH [16]. The work of Qureshi and colleagues, which included 323 patients with SAH, adds further support for these findings [26]. On examination of the medical records of those with SAH admitted to John Hopkins Medical Center between 1990 and 1997, an analysis of risk factors was performed using community-based controls matched for age, sex and ethnicity. More patients with SAH (46%) were smokers than controls (29%), providing an adjusted odds ratio (OR) of 5.2 (95% CI, 3.6–7.5) for current smokers.

**Table 2:** Smoking and the risk of subarachnoid haemorrhage: summary of studies

	**No. SAH Cases (No. in cohort)**	**No. of controls**	**Smoking Status**	**RR/OR**	**95% CI**	**Adjustments†**	**Matching**
**Case-Control Studies**							

Gill et al. [12] British Midlands, UK, 1989	208	573	Nonsmoker	1.00		Age, race, social class, alcohol consumption and treatment for hypertension	
			Current (Male)	4.52	2.4–8.4		
			Current (Female)	2.52	1.4–4.5		
							
Quereshi et al. [26] Maryland US, 2001	323	969	Never	1.0		Hypertension, diabetes, alcohol consumption.	Age, sex, and ethnicity.
			Current Smoker	5.2	3.6–7.5		
			Previous Smoker	4.5	3.1–6.5		
							
Isaksen et al. [16] Tromsø County, Norway, 2002	26	104	Never	1.00		Systolic and diastolic blood pressure, serum cholesterol, serum HDL, BMI, coffee consumption.	Age and sex.
			Former	2.13	1.04–4.39		
			Current	4.55	1.08–19.30		
							

**Cohort Studies**							

Kawachi et al. [18] Nurses' Health Study, U.S. 1993	108 (117006 females)	Rest of Cohort	Never Smokers	1.00		Age, hypertension, diabetes, BMI, high cholesterol, oral contraceptive use, HRT, age of smoking commencement, and follow-up period.	
			Past Smokers	2.26	1.16–4.42		
			Current Smokers	4.85	2.90–8.11		
			1–14/day	4.28	1.88–9.77		
			15–24/day	4.02	1.90–8.54		
			25–34/day	7.95	3.50–18.07		
			≥ 35/day	10.22	4.03–25.94		
							
Kurth et al. [19] U.S. Physicians' Health Study 2003	31 (22022 males)	Rest of Cohort	Never Smokers	1.00		Age, sex, and randomized treatment group	
			Ex-Smokers	0.79	0.37–1.68		
			Current (<20/day)	1.75	0.24–13.09		
			Current (≥ 20/day	3.22	1.26–8.18		

SAH, subarachnoid haemorrhage; RR, relative risk; OR, odds ratio; CI, 95% Confidence Interval; †adjustments, refers to adjustments made for either univariate or multivariate analysis. HDL, high density lipoprotein; BMI, body mass index; HRT, hormone replacement therapy.

In a cohort study Kawachi and colleagues found an adjusted relative risk of SAH of 4.85 (95% CI, 2.90–8.11) among current women smokers [18]. Importantly, these investigators also found that those smoking 1–14 cigarettes/day had an adjusted relative risk (RR) of SAH of 4.28 (95% CI, 1.88–9.77), which increased to 10.22 (95% CI, 4.03–25.94) in those smoking ≥ 35 cigarettes/day. They further reported a dose-response relationship between the daily number of cigarettes smoked and SAH (p < 0.0004). This provides evidence for a causal association between smoking and SAH.

The RR of SAH among former smokers is consistently less than that among current smokers [16,18,19,26]. In the Nurses' Health Study, women who ceased smoking were at less risk of SAH than those who continued to smoke, with RRs of 2.01 (95% CI, 1.12–3.61) and 4.96 (95% CI 3.13–7.87) respectively [18]. The investigators also reported that the RR of SAH among former smokers returned to the level of never smokers during the interval between 5 and 9 years following smoking cessation.

These studies demonstrate that smoking is a major independent risk factor for SAH. Importantly, this association is strengthened by the presence of a dose-response relationship. Of note is that ex-smokers are found to be at a higher risk of SAH than never smokers for many years after smoking cessation, but it appears that within 10 years their risk will return to that of a never smoker [18].

## Intracerebral Haemorrhage

In a meta-analysis of published studies of smoking and intracerebral haemorrhage (ICH), the pooled relative risk of ICH associated with smoking was 0.74 (95% CI, 0.56–0.98) [28]. However, the authors acknowledged that this result was heavily influenced by a single study conducted in neurological hospitals. There have been conflicting reports of the role of smoking in the development of ICH. Some investigators have reported that heavy smoking is associated with ICH [17,19], while others have reported no association between smoking and ICH (Table 3) [12,24,39].

**Table 3:** Smoking and the risk of intracerebral haemorrhage: summary of studies

	**No. ICH cases (No. in cohort)**	**No. controls**	**Smoking Status**	**RR/OR**	**95% CI**	**Adjustments†**	**Matching**
**Case-Control Studies**							

Gill et al. [12] British Midlands, UK, 1989	104	573	Non Smokers	1.00		Age, race, social class, alcohol consumption, and treatment of hypertension.	
			Current (Male)	1.82	0.9–3.7		
			Current (Female)	1.30	0.5–3.4		
							
Monforte et al. [24] Barcelona, Spain, 1990	24	48	Non-smokers	1.00		No adjustments.	Age and sex.
			Current smokers	1.52	0.41–5.57		
							
Jamrozik et al. [17] Perth, Australia, 1994	59	279	Never Smoker	1.00		Alcohol consumption, history of hypertension, diabetes, previous stroke or TIA, previous MI, adding salt to food, fish consumption > 2 per month.	Sex and age.
			Ex-smoker	1.11	0.43–2.85		
			Current (1–20/day)	3.17	0.92–11.0		
			Current (≥ 21/day)	9.84	2.09–46.4		
							
Thrift et al.[39] Melbourne, Australia, 1999	331	331	Never	1.00		Hypertension, cholesterol, BMI, previous cardiovascular disease, exercise, education level, diabetes, and alcohol.	Age, sex, and neighbourhood.
			Smokers	0.96	0.63–1.45		
			Ever Smokers	1.07	0.63–1.81		
			Current Smoker	0.89	0.56–1.42		
			Previous Smoker				
							
Woo et al. [40] Cincinatti, US, 2002	188 survivors	368	Non-smokers	1.0		No adjustments	
			Current Smokers	1.3	0.8–2.0	All ICH	
			Current Smokers	2.4	1.1–5.2	Lobar ICH	
			Current Smokers	0.9	0.5–1.6	Non-Lobar ICH	

**Cohort Studies**							

Kawachi et al. [18] Nurses' Health Study, US, 1993	53 (117006 Females)	Rest of Cohort	Never Smokers	1.00		Age, hypertension, diabetes, BMI, high cholesterol, oral contraceptive use, HRT, age of smoking commencement, and follow-up period.	
			Past Smokers	1.24	0.64–2.42		
			Current Smokers	1.24	0.64–2.42		
			1–14/day	1.68	0.34–5.28		
			15–24/day	2.53	0.71–6.05		
			≥ 25/day	1.41	0.39–5.05		
							
Kurth et al. [19] US Physicians' Health Study, 2003	108 (22022 Males)	Rest of Cohort	Never Smokers	1.00		Age, exercise, parental history of MI before age 60, alcohol consumption, and randomized treatment group	
			Past Smokers	0.80	0.54–1.20		
			Current (<20/day)	1.60	0.50–5.07		
			Current (≥ 20/day)	2.06	1.08–3.96		

ICH, intracerebral haemorrhage; RR, relative risk; OR, odds ratio; 95% CI, 95% Confidence Interval; †adjustments, refers to adjustments made for either univariate or multivariate analysis. TIA, transient ischaemic attack; MI, myocardial infarction; BMI, body mass index; HRT, hormone replacement therapy.

The most recent published data on smoking and intracerebral haemorrhage comes from the Physicians' Health Study, which included 108 cases of ICH [19]. The investigators found that the RR for ICH among current smokers was 1.98 (95% CI 1.07–3.65) when compared to never smokers. This RR was adjusted for age, alcohol consumption, exercise, parental history of myocardial infarction under 60 years and randomised antioxidant treatment. A non-significant dose-response trend was noted with those smoking <20 cigarettes/day having a 1.6–fold increase (RR 1.6, 95% CI, .50–5.07) in the risk of intracerebral haemorrhage, with the risk increasing to a 2.1–fold increase (RR 2.06, 95% CI, 1.08–3.96) in those smoking ≥ 20 cigarettes/day.

Another study that somewhat supports the findings of Kurth and colleagues involved a prospective study of haemorrhagic stroke patients in the US [40]. Although the investigators found smoking was not an independent risk factor for haemorrhagic strokes overall (OR 1.3, 95% CI, 0.8–2.0), this finding was limited by those with non-lobar haemorrhage [40]. In contrast the association between smoking and lobar haemorrhages was more than twofold (OR 2.4, 95% CI, 1.1–5.2).

In contrast to the previously mentioned findings, numerous investigators have found no association between smoking and ICH. In a large sample of patients with ICH (331) and using case-control methodology, Thrift and colleagues found no increased risk of intracerebral haemorrhage associated with smoking (adjusted OR, 1.07, 95% CI, 0.63–1.81) [39]. Importantly, the cases and controls in this study were matched for age (± 5 years), sex, and neighborhood of residence (the latter allowing some degree of matching for socioeconomic status). In addition, the potentially confounding effects of hypertension, cholesterol, body mass index, previous cardiovascular disease, exercise, education level, diabetes, and alcohol consumption were adjusted for in the analyses.

The results of 13 studies, in which the contribution of smoking in the risk of ICH was investigated, were included in a recent meta-analysis [41]. The authors found that the risk of ICH with smoking was 1.31 (95% CI, 1.09–1.50). However, this study was limited by the fact that no adjustments could be made by potentially confounding factors, and so the independence of smoking as a risk factor could not be established. Therefore, the contribution of smoking to the risk of ICH is still in doubt.

## Passive Smoking and the Risk of Stroke

Beyond the damage that active smoking may cause an individual are the adverse effects that inhaling environmental tobacco smoke (ETS) from the sidestream smoke of a burning cigarette may cause others. This smoke contains high concentrations of CO, nicotine and carcinogens [42]. In some cases these combustion products are of a higher concentration in sidestream smoke than in the mainstream smoke inhaled by the smoker [42]. As many investigations have been made into the role of active smoking in the development of stroke, it is interesting to find that only a small number of studies have been focused on the role of ETS in the pathogenesis of stroke.

The first examination of the link between exposure to ETS and the development of stroke was conducted by Lee and colleagues using a hospital based case-control study [43]. The study included non-smokers whose spouses were current smokers with lung cancer. The non-smoking spouses (n = 55) were then matched to controls with non-smoking spouses (n = 254). No significant increase in the risk of stroke was found among smokers' spouses (RR 0.90, 95% CI, 0.53–1.52) when compared to non-smokers' spouses. In contrast to these findings, in a case-control study involving 452 cases, You and colleagues reported a borderline association between ischaemic stroke and exposure to ETS via a spouse in non-smokers (OR 1.70, 95% CI, 0.98–2.92) [33].

The most recent study on passive smoking and stroke was a population based case-control study conducted in New Zealand [8]. The study involved 521 cases of first ever stroke and 1,800 community-based controls. The authors defined passive smokers as those exposed to smoking via a household member who regularly smoked in their presence or a co-worker who smoked indoors in their presence for more than one of the past 10 years. After adjusting for potentially confounding factors such as sex, hypertension and diabetes, the odds ratio of developing stroke associated with exposure to ETS was 1.82 (95% CI, 1.34–2.49). This provides some evidence that non-smokers who are exposed to ETS have a greater risk of developing stroke than those not exposed to ETS.

In support of these findings, potential mechanisms whereby ETS may contribute to the pathogenesis of stroke have been identified. The Atherosclerosis Risk in Communities (ARIC) study investigators presented findings linking ETS to the development of carotid atherosclerosis [44]. After controlling for demographic, life-style and other risk factors, intimalmedial thickening was found to be greater in non-smokers who were exposed to ETS, than non-smokers who were not exposed to ETS. In addition, the rate of progression of intimalmedial thickening was significantly greater among non-smokers exposed to ETS (p = 0.003). Furthermore, Kiechl and colleagues found non-smokers exposed to ETS had a greater risk of developing early (OR 1.3, 95% CI, 1.0–1.8) and advanced atherosclerosis (OR 1.5, 95% CI, 1.0–2.2) than non-smokers not exposed to ETS [45].

Overall, data from the few studies conducted on exposure to ETS and the development of stroke, together with data concerning ETS and atherosclerosis, provide some support for the notion that ETS can lead to the development of stroke. To clarify the role of ETS in the development of stroke, studies must be designed to determine whether altering the amount or duration of ETS changes the rate of stroke within a population. More evidence is needed on the role of ETS in the development of stroke. An examination of the effect of ETS by stroke subtype may better evaluate the relationship between this potentially modifiable risk factor and stroke.

## Cigarette Smoking in the Pathogenesis of Stroke

Smoking appears to be involved in the pathogenesis of stroke via two mechanisms. First, smoking can cause direct damage to the vasculature, altering both its architecture and function [44-54]. Second, smoking has effects on haemodynamic factors within the circulation [53,55-64]. By discussing the implications of these alterations in function by stroke aetiology, the role of smoking in the pathogenesis of stroke is better elucidated. Two distinct types of stroke will be discussed, cerebral infarction and haemorrhagic stroke (including ICH and SAH).

### Smoking and the development of cerebral infarction

Cerebral infarction occurs due to a disruption of the blood supply to the cerebral arteries. This can be a consequence of either occlusion of the cerebral vessels themselves or due to occlusion of the carotid arteries. There are a number of ways in which this occlusion can occur including artery occlusion by atherosclerosis and its associated plaques and thrombi or via emboli from a ruptured atherosclerotic plaque.

Smoking is a major factor in the development of atherosclerosis. In the ARIC study current smokers were found to have the greatest progression of atherosclerosis over time, at a rate 50% greater than in non-smokers [44]. Furthermore, it was found that ETS exposure was associated with an approximately 20% greater rate of atherosclerosis progression than for non-smokers not exposed to ETS. Similar findings have been reported by others [48].

Smoking is believed to induce the development of atherosclerosis by initiating endothelial injury, presumably due to either the production of oxygen radicals or via direct toxic effects of cigarette smoke constituents. Even brief exposure to cigarette smoke has been found to activate leukocytes, stimulating the release of the pro-coagulant, von Willebrand Factor (vWF) and causing endothelial damage [56]. This initiates a cascade of inflammatory mechanisms that result in atherosclerosis.

The mechanisms by which endothelial dysfunction and a reduction in dilatory ability occur were further investigated by Fang and colleagues in the cerebral arteries of rats [49]. They reported that nicotine infusion reduced the dilatory ability of pial vessels to nitric oxide synthase (NOS) dependant agonists. In addition, this response was inhibited by administration of superoxide dismutase. These findings provide evidence that nicotine has its effects via the formation of oxygen radicals. The authors speculated that this oxygen radical induced impairment of dilation is important in the occurrence of vascular dysfunction after exposure to cigarette smoke.

Studies on arteries of human smokers have found similar results to Fang and colleagues. Poredôs and colleagues reported data on the intima-media thickness (IMT) of smokers compared with non-smokers, with IMT being used as a measure of atherosclerotic change [65]. The investigators found that those who smoked had a significantly greater IMT than non-smokers. In addition, Celermajer and colleagues demonstrated that the arteries of smokers had diminished or absent flow-mediated dilation, giving evidence of endothelial dysfunction [66].

The ramifications of the reduction in dilatory ability following smoking may place smoking individuals at a higher risk of cerebral ischaemic events in two ways. First, due to both a reduced ability to respond to alteration in perfusion pressure, and second, due to lack of distensibility of the arterial wall as a result of endothelial dysfunction. The notion that this lack of distensibility of the arterial wall may increase the risk of atherosclerotic plaque rupture, leading to emboli and subsequent infarction, was presented by Kool and colleagues [52]. They found that blood pressure and heart rate were significantly increased following smoking, whereas carotid artery distensibility was significantly decreased. It was postulated that the increased wall stiffness, blood pressure and heart rate could cause an increase in load on the vessel wall, which could lead to plaque rupture and acute ischaemic events. This is particularly relevant in light of evidence that smokers have increased levels of atherosclerosis.

Studies conducted on the reactivity of the cerebral vessels of smokers are particularly pertinent to the role of smoking in the development of stroke. Terborg and colleagues investigated the reactivity of the cerebral circulation in smokers to hypercapnia using near-infrared spectroscopy and transcranial Doppler sonography [67]. It was found that at rest smokers have normal autoregulatory ability, yet immediately after smoking their vasomotor reactivity to hypercapnia was impaired. This was due to a reduced vasodilatory ability reflecting endothelial dysfunction. It was suggested that these acute reversible effects of cigarette smoking could place smokers at an enhanced stroke risk, presumably due to the reduced ability of the cerebral circulation to response to decreases or disruptions in perfusion pressure in this period immediately following smoking.

In addition to the atherosclerotic changes that cigarette smoking induces in the vessel wall, smoking also has detrimental effects on the coagulant/anticoagulant balance throughout the vascular system of smokers. This imbalance predisposes these individuals to thrombosis and therefore an increased risk of cerebral infarction. Hioki and colleagues investigated the effects of smoking on thrombin generation, which is important in the conversion of fibrinogen to fibrin in the coagulant cascade [57]. It was evident from their findings that even when not smoking, current smokers had significantly elevated levels of thrombin. These levels were seen to increase further immediately after smoking. Examination of serum of smokers also reveals that smokers have significantly increased fibrinogen and white blood cell counts when compared to non-smokers [68], giving further credence to the suggestion that smokers are more prone to coagulation and thrombosis than non-smokers. Investigations on the effect of nicotine on haemostatic functions have found that nicotine increases the production of the tissue plasminogen activator (tPA) inhibitor (plasminogen activator inhibitor-1, PAI-1). The presence of tPA in the circulation is important for fibrinolysis. Its inhibition is regulated by PAI-1 from both platelet and endothelial sources, which binds to fibrin strands causing the inactivation of tPA. This ultimately results in a reduction in fibrinolysis and therefore thrombus formation. A study exposing human brain endothelial cells to nicotine is particularly relevant to the discussion of smoking and stroke. In this study Zidovetski and colleagues discovered that the administration of nicotine to these cells resulted in an increased production of PAI-1 [63].

Human studies show similar findings, with long-term smokers shown to have significantly increased levels of PAI-1 when compared to non-smokers [61]. In addition, the ratio of tPA to PAI-1 was found to be lowest in smokers, suggesting that smokers have the lowest potential for fibrinolytic activity that may lead to a greater tendency for thrombus among smokers. The authors noted the apparent close association between tPA and PAI-1 levels and suggested that there is most probably mechanisms ensuring a balance of the activator and inhibitor. In smokers it would appear that this balance is not maintained and this may predispose these individuals to pathological conditions such as stroke. Investigations into tPA and PAI-1 levels in stroke patients reveal that patients have significantly elevated plasma levels of both tPA and PAI-1, along with increased active PAI-1 [64]. It was also found that there was a significant increase in tPA: PAI-1 complexes, suggesting that PAI-1 is inhibiting active tPA. This provides evidence that patients with stroke may indeed have decreased fibrinolytic activity resulting in an increased risk of thrombosis and therefore cerebral infarction.

These investigations demonstrate that cigarette smoking induces both short-term and long-term alterations in haemodynamic variables, and can also alter arterial wall architecture resulting in atherosclerosis. In turn these alterations can result in occlusion of vessels due to stenosis within the carotid region and emboli from a ruptured atherosclerotic plaque, increasing the risk of a cerebral infarction. The reduction in the risk of cerebral infarction following smoking cessation may be due to the resolution of acute pathological changes caused by smoking such as alterations in haemodynamics, rather than a reversal of more chronic changes such as atherosclerosis. However, this is speculative as the effects of smoking cessation on these variables has not been investigated.

### Smoking and the development of haemorrhagic stroke

The role of smoking in the pathogenesis of haemorrhagic stroke has been less investigated. This is surprising given the strong association found between smoking and SAH in particular [6,12,16,18,19,26,28]. Cerebral haemorrhage occurs due to a rupture of a cerebral artery causing an influx of blood into either the subarachnoid space (in SAH) or into the brain parenchyma (in ICH).

SAH is known to be due to the rupture of an artery and in many cases the rupture of a saccular aneurysm [69]. Importantly, it has been found that smokers have a greater amount of aneurysms that are also larger in size than those in non-smokers [70,71]. ICH is generally associated with the rupture of small arteries within the brain parenchyma. As with cerebral infarction, it would appear that it is structural damage to the arterial wall that leads to the increased risk of haemorrhage. Aneurysms and/or rupture of cerebral arteries occur due to a weakening of the vessel wall. In the case of aneurysm it has been proposed that the degradation of elastin within the blood vessel wall may weaken the wall, making it susceptible to dilatation at points of high flow disturbance such as the branching of arteries within the Circle of Willis [70]. This degradation of elastin may be due to a reduction of the activity of protease inhibitors, namely α_1 _antitrypsin. Smoking is known to inactivate α_1 _antitrypsin via peroxynitrates and •OH compounds found within cigarette smoke [50]. Furthermore, examination of smokers has found increased elastase degradation products in their urine [72]. Tartara and colleagues suggested that a defect in α_1 _antitrypsin activity may cause an imbalance between proteolytic enzymes and systemic inhibitory capacity; in turn there would be an increase in collagen turnover resulting in a weakening of the arterial wall [73]. This might result in the progression of an aneurysm or also rupture of aneurysm, resulting in SAH, or the decreased strength of vessel walls could result in intracerebral arterial rupture resulting in ICH. In support of this hypothesis are data showing that smoking patients with SAH have significantly reduced levels of α_1 _antitrypsin [73].

The potential implications of these structural alterations in the arterial wall of smokers may also lead to an increased risk of haemorrhage due to the alteration in blood pressure caused by smoking. A sharp rise in blood pressure has been observed immediately after smoking and can last for up to three hours [22,52]. Juvela and colleagues proposed that these rises in blood pressure could contribute to the rupture of aneurysms or even small intracerebral arteries [74].

The data relating to acute changes due to smoking, such as blood pressure surges, teamed with data on chronic smoking related changes, such as arterial wall degradation, provide a hypothesis for the role of smoking in the development of haemorrhagic stroke. Furthermore, it is evident that the cessation of smoking may lower haemorrhagic stroke risk due to the elimination of acute effects such as blood pressure surges. However, the chronic effects of smoking such as arterial wall degradation may remain. This may explain why although the risk of haemorrhagic stroke declines with smoking cessation, it does not revert to that of an individual who has never smoked [16,18,19,26].

## Conclusion

Smoking is an established risk factor for both cerebral ischaemia and subarachnoid haemorrhage. However, its role in ICH is less clear. There is supporting evidence for a causal relationship between smoking and CI or SAH. This is supported by reports of a dose-response relationship between smoking levels and these two types of stroke, as well as a return to never smoking risk levels with increased time since quitting smoking. Furthermore, the biological plausibility of these findings provides further evidence for the role of smoking in these diseases.

Establishing smoking as a significant risk factor for CI and SAH is important, as this is a modifiable risk factor. The evidence that among smokers who cease smoking the risk of both of these forms of stroke returns to never smoking levels over time indicates that people who cease smoking can benefit in an important way. Prevention still remains the most effective way of reducing the impact of stroke.

## Competing interests

The authors declare that they have no competing interests.
